# Visual Sensor Fusion Based Autonomous Robotic System for Assistive Drinking

**DOI:** 10.3390/s21165419

**Published:** 2021-08-11

**Authors:** Pieter Try, Steffen Schöllmann, Lukas Wöhle, Marion Gebhard

**Affiliations:** Group of Sensors and Actuators, Department of Electrical Engineering and Applied Sciences, Westphalian University of Applied Sciences, 45877 Gelsenkirchen, Germany; steffen.schoellmann@w-hs.de (S.S.); lukas.woehle@w-hs.de (L.W.); marion.gebhard@w-hs.de (M.G.)

**Keywords:** human robot interaction, assistive robotics, sensor fusion, localization, computer vision, visual servoing, autonomous robotic system

## Abstract

People with severe motor impairments like tetraplegia are restricted in activities of daily living (ADL) and are dependent on continuous human assistance. Assistive robots perform physical tasks in the context of ADLs to support people in need of assistance. In this work a sensor fusion algorithm and a robot control algorithm for localizing the user’s mouth and autonomously navigating a robot arm are proposed for the assistive drinking task. The sensor fusion algorithm is implemented in a visual tracking system which consists of a 2-D camera and a single point time-of-flight distance sensor. The sensor fusion algorithm utilizes computer vision to combine camera images and distance measurements to achieve reliable localization of the user’s mouth. The robot control algorithm uses visual servoing to navigate a robot-handled drinking cup to the mouth and establish physical contact with the lips. This system features an abort command that is triggered by turning the head and unambiguous tracking of multiple faces which enable safe human robot interaction. A study with nine able-bodied test subjects shows that the proposed system reliably localizes the mouth and is able to autonomously navigate the cup to establish physical contact with the mouth.

## 1. Introduction

The World Health Organization estimates that between 785 (15.6%) to 975 (19.4%) million people of the global population are affected by disability. Of this group, between 110 to 190 million experience significant functional restrictions [1]. These can range from seeing and hearing restrictions to the paralysis of limbs. As a result, these people have problems to independently perform activities of daily living (ADL). Tetraplegia is a form of paralysis in which all four limbs are paralyzed. People with tetraplegia need a wheelchair and are unable to perform many ADL by themselves due to loss of hand function. They are dependent on continuous support by human caregivers and greatly benefit from supporting systems that enable them to perform tasks independently.

Assistive robotics is a research field which aims to support people with disability and people in need of assistance e.g., people with tetraplegia, with robotic systems. Assistive robots are designed to perform physical tasks in the context of ADLs to improve well-being and independence in performing ADLs. These robots are deployed on wheelchairs and in homes to interact with the user. The human-machine interface (HMI) is a major challenge of this research field because most members of the target group have limited mobility and are not able to use traditional input devices like joysticks and buttons. Several approaches utilized electrode caps for brain-machine interfaces (BMIs) and wheelchair-mounted chin joysticks as an input device to control robotic systems. Disadvantages of this approach are the required infrastructure and the demanding task of controlling a robot arm in 3-D space. A solution employed in more recent approaches are semi-autonomous systems that need a minimal amount of input from the user to perform assistive robotics tasks. This approach requires precise motion control of the robot and awareness of the environment to prevent accidents. Therefore, robust sensing systems and sophisticated control algorithms are needed to develop assistive robotic systems for people with tetraplegia.

Among the several applications of assistive robotics independently eating and drinking are especially desired. This is shown in a survey of potential users [2] where eating and drinking were chosen as high priority ADLs for assistive robotics. Independently drinking is especially desired due to the regularity of the task. A major challenge is precise navigation, since eating and drinking involve precise movements in the facial area and physical contact with tools like cups and spoons.

There have been multiple approaches (see Section 2) to design a robotic system that enable people with tetraplegia to drink independently. This application is called assistive drinking and uses a robot arm to manipulate a drinking cup which greatly increases independence in daily life, as drinking would otherwise require the help of a human assistant. As a general description, assistive drinking comprises of three tasks that the robot arm has to perform:

1. filling the cup with a beverage and grasping it with the gripper of the robot arm
2. localizing the mouth and navigating the robot-handled cup to the mouth
3. tilting the cup to let the user drink the beverage

The described procedure allows the user to drink from a robot-handled cup with direct physical contact between the cup and the user’s mouth. This approach requires precise navigation of the robot arm for safe operation. Since the target group cannot use traditional input devices like joysticks or buttons, alternative interfaces based on head movement or gaze are necessary to interact with the robot for assistive drinking. In previous work [3,4] a head motion-based interface is presented which allows a person with tetraplegia to control a cursor on a graphical user interface (GUI) as well as control a robot arm or a wheelchair. This interface enables manual control of all six degrees of freedom (DOF) of a robot arm as well as macros which contain sets of instructions to perform tasks like pouring a drink in a cup independently. However, manual control of a robot arm requires high levels of mental and physical effort. Therefore, an autonomous implementation of assistive drinking is highly desirable to reduce the mental and physical load on the user. An autonomous assistive drinking procedure could be selected and started with the head motion-based interface.

In this paper a sensor fusion algorithm for localizing the user’s mouth and a robot control algorithm are presented to autonomously perform the second task of assistive drinking (localization of the mouth and navigating the cup to the mouth). The proposed algorithms were successfully implemented in a robotic system which consists of a robot arm and a visual tracking system on the robot’s gripper for tracking and localizing the head and mouth of the user. In addition, a capacitive sensor was incorporated on the rim of the cup to detect physical contact with the user. The entire system is contained on the robotic arm and does not require any infrastructure. The proposed system enables the robust localization of the mouth and precise navigation of the robot-handled cup to perform assistive drinking autonomously while providing features to achieve functional safety.

The main contribution of this work is the visual tracking system and the robot control algorithm. The visual tracking system uses a 2-D camera and a time of flight (TOF) single point distance sensor to track faces and localize the user’s mouth for navigation purposes. The proposed sensor fusion algorithm uses computer vision and projection to combine data of both visual sensors. This approach enables the detection and correction of alignment errors of the distance sensor to achieve robust and precise localization of the mouth. The proposed robot control algorithm uses visual servoing and data from the sensor fusion algorithm to correct the alignment of the distance sensor in real-time and simultaneously navigate the cup to the mouth. Additional features of the visual tracking system are unambiguous tracking of multiple faces to prevent a mix-up in case multiple faces are visible in the camera image and the detection of head turns which the user can use as an abort command to stop the robot.

## 2. State of the Art

A major challenge of assistive robotics i.e., assistive drinking, is the human robot interaction and autonomous operation of the robotic system to provide a high level of safety and usability. A study showed that autonomous operation of the robot is desired, as it can be very demanding to control a robot arm in 3D-space [5].

Multiple approaches have been presented to perform assistive robotics applications with a robot arm. The FRIEND system uses a wheelchair mounted robot arm (WMRA) which allows a person with tetraplegia to work as a librarian [6]. The HMI consists of a set of buttons and a chin joystick for head motion control supported by a GUI on a monitor. This setup is used as a head operated computer mouse to control the wheelchair and WMRA with the GUI. Previous versions also addressed the drinking task, but were restricted to drinking with a straw for safety reasons [7]. AMiCUS is a human robot interface that enables people with tetraplegia to control a robot arm with multiple DOF in real-time solely using head motion, empowering them to perform simple manipulation tasks independently. It is based on a single attitude heading reference system that is worn on the head [3,4].

Another approach to assistive drinking uses BMIs for controlling a robotic arm to drink without a straw [8]. An externally mounted RGB-D camera is used to estimate the user’s mouth by applying a golden-ratio approach on the detected face, thus bypassing issues of face occlusion by the cup and the robot. The user controls the scenario by giving GO-signals via the BMI and can thereby control the speed of the procedure in seven discrete predefined steps.

Other hands-free HMIs concepts were investigated. An eye gesture based system [9] and BMI based systems [10,11] were developed to allow people with tetraplegia to control a robot arm with seven degrees of freedom.

An underlying problem of BMI-based systems are the head mounted sensors that have to be positioned on people with tetraplegia by attendees. As a solution, Goldau et al. [12] presented a system for assistive drinking where all sensor components are contained on the robot arm. It consists of an RGB-D camera-based tracking system [13] and a multi-sensor system for force control [14]. The RGB-D camera system estimates the user’s head pose to navigate the robot arm while the multi-sensor system is used to establish physical contact and control the robot’s speed depending on the distance between the cup and mouth. In addition to navigation, the head pose is used to implement an abort command for emergencies which is triggered when the user does not face the robot directly. The multi-sensor system measures the contact force on the cup rim with a force sensing resistor and the distance between the mouth and cup with three IR sensors that are calibrated on a doll head. However, varying head shapes and misalignment of the IR sensors result in large errors that make the distance measurement unreliable for precise positioning of the cup. Furthermore, the two IR sensors mounted on the robot’s opposing fingers can interfere with the user’s face.

With recent technological advances in miniaturized TOF distance sensors and computer vision algorithms, this work proposes a sensor fusion algorithm and a robot control algorithm to utilize commercially available sensors for precise localization of the face and mouth in assistive drinking with an autonomous robotic system. The proposed sensor fusion algorithm is implemented in a visual tracking system that consists of a 2D-camera and a miniature TOF distance sensor, which are mounted on the robot’s wrist. The visual tracking system uses the TOF sensor to measure the distance to the mouth which is transformed in regards to the robot’s coordinate system to get the location of the mouth. In order to ensure that the TOF sensor is oriented towards the mouth, the sensor fusion algorithm uses a projection of the distance measurement and location data of facial features, obtained from computer vision algorithms, to constantly evaluate if the distance sensor is aimed at the mouth as well as how far the aim deviates from the mouth. The deviation from the mouth is send to the proposed robot control algorithm which uses visual servoing to autonomously correct the orientation of the robot-mounted sensor and point it at the user’s mouth. The proposed data fusion enables a robot to reliably localize the mouth with a TOF sensor and correct misalignment automatically which is a crucial feature to achieve robust localization of the mouth. Additional features of the proposed system include the unambiguous tracking of multiple faces to prevent unexpected behavior and the detection of a head turn triggered abort command which are implemented in the computer vision algorithm.

## 3. Visual Sensor Fusion Based Autonomous Robotic System

This work proposes a visual tracking system based on the sensor fusion of multiple optical sensors as well as a robot control algorithm for autonomous navigation in assistive drinking. The visual tracking system uses commercially available sensors to localize facial features for performing assistive drinking with physical contact. The tracking system combines 2-D camera images and distance measurements from a single point TOF sensor with a narrow field of view (FOV) (approx. 7°) to reliably localize facial features and measure the distance between the robots end-effector and the mouth. Furthermore, a robot control algorithm is presented that uses visual servoing to navigate the robot arm and correct misalignment of the tracking system simultaneously.

**Figure 1:**
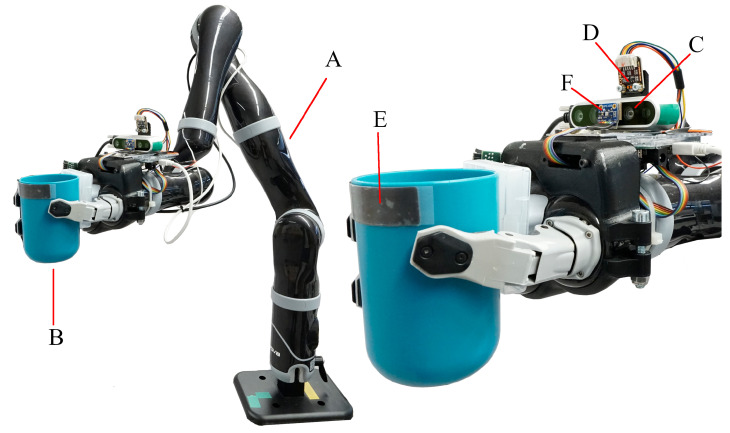
The robotic system. (A) Kinova Jaco Robot Arm (B) Drinking Cup (C) Intel RealSense D435 Camera (D) VL53L1X Distance Sensor (E) Tacterion Plyon Force and Capacitive Sensor. (F) Environmental Sensor (part of our previous work [15]) which is not used in this system.

The performance of the proposed tracking system and navigation algorithm is evaluated in two experimental studies with nine able-bodied subjects. In the first study the overall performance of the proposed system is evaluated by determining the success rate of performing the cup delivery. The cup delivery involves the navigation of the cup from a starting position to the mouth of the user. The second study evaluates the reliability of the implemented abort command as well as the required range of motion for safe operation. The robotic system is shown in Figure 1.

### 3.1. Robotic System for Assistive Drinking

Figure 1 shows the robotic system with all components. The proposed setup uses the Jaco^®^robot arm *(A)* from Kinova^®^which has seven DOF. It is designed as a WMRA and is characterized by a light-weight design and low power consumption. Though, it lacks dedicated force and torque sensors. A 3-finger gripper is assembled on the robot arm to grasp the drinking cup *(B)*. A stripe shaped sensor *(E)*, assembled below the rim of the drinking cup, is capable of measuring force and capacitance. This sensor is used to detect physical contact with the user’s lips and is presented in detail in our previous work [15]. The visual tracking system is fitted on a mount on the wrist of the robot arm and consists of a camera *(C)* and a distance sensor *(D)*.

An Intel^®^i7 6700T based computer is used to control the robotic system. It runs on Linux 14.04 with the Robot Operating System (ROS) to control the robot arm and perform computation tasks like signal processing and computer vision. All algorithms and software components are written in C++ and implemented in ROS. The visual tracking system is used to localize the user’s mouth for navigating the drinking cup held in the 3-finger gripper. This setup is designed to perform assisted drinking with a user sitting approximately 10 cm to 40 cm in front of the robot arm.

### 3.2. Visual Tracking System

In this work a sensor fusion algorithm is proposed for reliable localization of facial features in assistive drinking using a visual tracking system. The tracking system consists of a single-point TOF distance sensor with a narrow FOV and a 2D-camera. The position of the mouth is estimated by measuring the distance to it with the distance sensor. Further transformation can be used to calculate the 3D coordinates of the mouth. As described in Section 2, this approach usually suffers from severe measurement error when the distance sensor is misaligned by movement of the user or the robot arm. However, our approach uses a camera and the proposed sensor fusion algorithm to evaluate if the distance sensor is correctly pointed at the mouth and calculate the deviation of the desired orientation to achieve robust and precise localization. This is achieved, by projecting a given distance measurement into the camera image using a rigid transformation and extracting the location of facial features in the camera image. The location of the mouth and the projection of the distance measurement are then compared to evaluate if the distance measurement corresponds to the mouth and how far the measurement deviates from the desired point. The deviation is send to the robot control algorithm which uses a closed loop controller to correct the orientation of the sensor. This approach yields precise and robust alignment of the tracking system for reliable localization of the mouth for navigation purposes.

**Figure 2:**
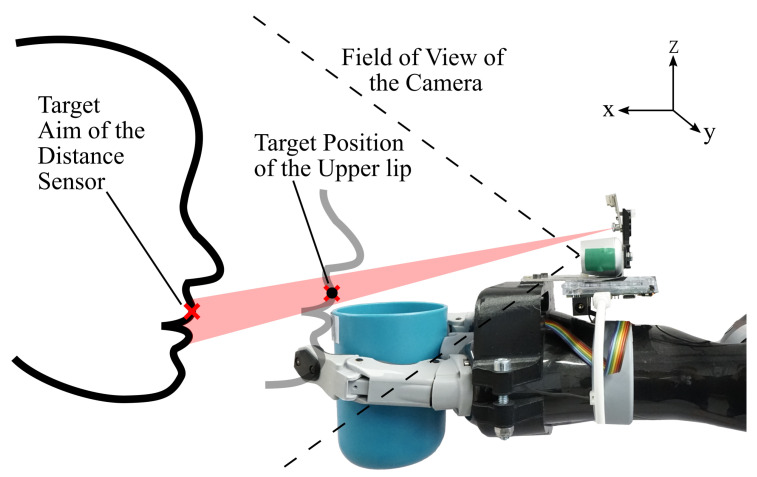
Physical setup of the visual tracking system. The dotted line represents the field of view of the camera. The red ray illustrates the field of view of the distance sensor. The black dot and transparent face illustrate the target position of the cup. The red cross represents the point where the distance sensor is supposed to be aimed at to measure the distance to the mouth.

Figure 2 shows the concept of the tracking system. The tracking system is assembled on the wrist of the robot arm and is oriented towards the gripper. This mounting position was chosen to minimize interference with the user’s face and to allow for uninterrupted visual contact with the user’s face and mouth during human robot interaction. The distance sensor is adjusted to point slightly above the cup rim for measuring the distance to the mouth. In particular, the distance sensor measures the distance to the upper lip, marked with a red cross in Figure 2, as it stays in line of sight with the distance sensor opposed to the lower lip which is occluded by the cup rim. In the target position the cup rim is positioned between the lips and in physical contact with the lower lip. The upper lip is above the cup rim, illustrated as a black dot in Figure 2, where it is still visible to the tracking system.

Our setup of the tracking system uses the Intel^®^RealSense™D435 RGB-D camera with three camera modules. As the tracking system requires a regular 2D camera image, only one of the three camera modules is enabled. Of the three camera modules the central infrared camera module is chosen for this system due to its superior FOV (100.6°), resolution (1280 × 800), and shutter type (global shutter). The infrared module produces images which are similar to greyscale images from an RGB camera. For the distance sensor, the VL53L1X TOF distance sensor from STMicroeletronics is used in this system for robust and reliable distance measurement. This distance sensor features an electrically adjustable FOV which is achieved with an adjustable 16 × 16 single photon avalanche diode (SPAD) receiver matrix. In this setup, the sensor is set to use a 4 × 4 SPAD matrix which reduces the FOV to approximately 7°.

**Figure 3:**
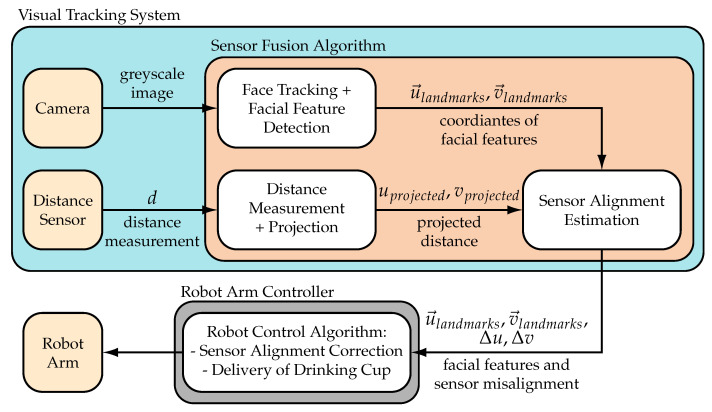
Block diagram showing the components of the robotic system for autonomous assistive drinking. The robotic system includes the robot arm, visual tracking system, and robot controller. The tracking system consists of the two hardware sensors and three software components. Another necessary component is the sensor alignment correction of the robot arm controller which together enable robust localization of the mouth.

The data pipeline of the proposed system is depicted in Figure 3 as a block diagram. Data from the sensors is processed by the sensor fusion algorithm in three software blocks and send to the robot controller where a closed loop controller autonomously corrects the alignment of the tracking system. The camera images are processed by the Face Tracking Block which tracks individual faces and basic facial features with a facial feature detector. The bounding box of the face, the coordinates of the facial features, and an estimation of the head orientation are send to the Sensor Alignment Estimation Block. The Distance Measurement Projection Block projects distance measurements to image coordinates using transformations and the pinhole camera model. The image coordinates of the projected distance measurement are also send to the Sensor Alignment Estimation Block where the image coordinates of the distance measurement and the desired location (image coordinates of the upper lip) are compared. The deviation Δu, Δv of the two points is send to the Robot Control Block. It uses visual servoing methods to correct the orientation of the sensor by moving the robot’s wrist. The proposed algorithm is able to run in real-time and enables robust tracking of the mouth with continuous correction of the sensor orientation. The software components, depicted in Figure 3 are described in detail in the following chapters.

#### 3.2.1. Face Tracking and Facial Landmark Detection

In this work a modified version of the face detection algorithm from [16] is proposed which is optimized for localization and human robot interaction. The main task of the face tracking algorithm is the unambiguous tracking of faces, the localization of facial features, and the estimation of the head orientation for the yaw and roll axis. The face tracking algorithm provides the robotic system with crucial data for the detection of the user, evaluation of the distance sensor alignment, and navigation of the robot-handled cup. In addition, it enables the user to stop the robot arm with an abort command by turning their head which is a crucial feature especially in unpredictable situations. The face tracking algorithm is able to run in real-time to respond to arbitrary movement of the user. In this setup, the algorithm is run 60 times per second which corresponds to the frame rate of the camera. The methodology proposed within this work is to combine fast detection algorithms with a decision tree structure for real-time tracking of faces and their facial features in a video stream. A key aspect is the temporal coherence contained in a video stream which allows the tracking algorithm to re-detect faces frame by frame for robust tracking. The optimization lies in the application oriented approach for face tracking which includes the addition of a facial landmark detector for the localization of facial features and a profile face detector for robust tracking even when a face is turned away from the camera. The computer vision algorithm is implemented in C++ with the open source computer vision libraries OpenCV [17] and dlib [18].

**Figure 4:**
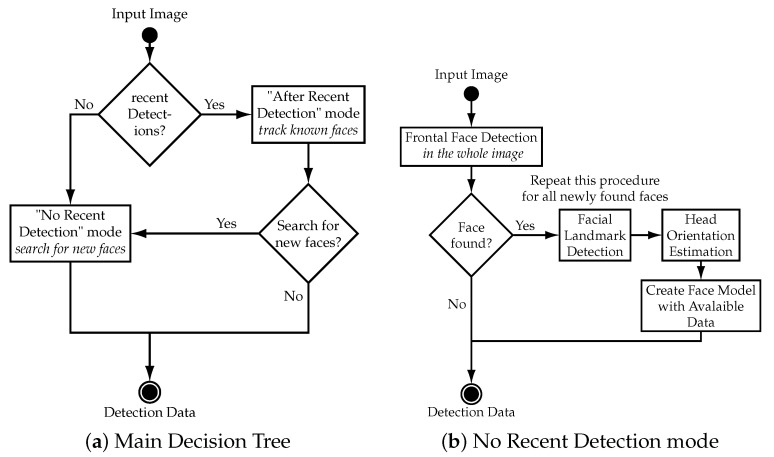
Decision tree of the main face tracking algorithm and “No Recent Detection” mode. The rectangle shapes illustrate tasks of the algorithm and the diamond shapes symbolize decisions.

**Figure 5:**
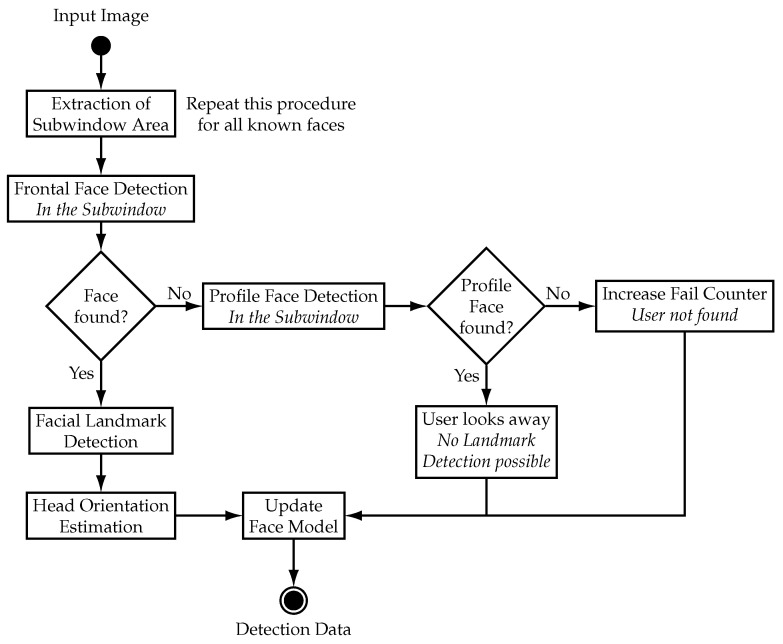
Decision tree of “After Recent Detection” mode. The rectangle shapes illustrate tasks of the algorithm and the diamond shapes symbolize decisions.

The main structure of the tracking algorithm is a decision tree that uses two different modes. The main decision tree is illustrated in Figure 4a and is called every time a new camera image is available. Upon receiving a new camera image, the algorithm uses either the “No Recent Detection” mode or the “After Recent Detection” mode, depending on recent face detection events. The “No Recent Detection” mode is used either at the start of a tracking session or when no face was visible for a certain amount of time. It searches the whole image for faces and collects data of every face it finds. This data is saved in an array called detection data which is preserved between calls of the algorithm. The “After Recent Detection” mode is used instead of the “No Recent Detection” mode when data of a face exists in the detection data array. This is the case either when the last iteration of the algorithm found a face with the “No Recent Detection” mode or if faces are being tracked by the “After Recent Detection” mode. The “After Recent Detection” mode tracks all known faces by searching in the vicinity of the last known position of the face to redetect it. This approach reduces computational effort when faces are tracked by reducing the area of the image that has to be processed. However, the “After Recent Detection” mode is not able to find new faces in the video stream. Therefore, the “No Recent Detection” mode is called periodically to check if any new face appeared in the video stream. The “No Recent Detection” mode is shown in Figure 4b and the “After Recent Detection” mode in Figure 5 respectively.

**Figure A1:**
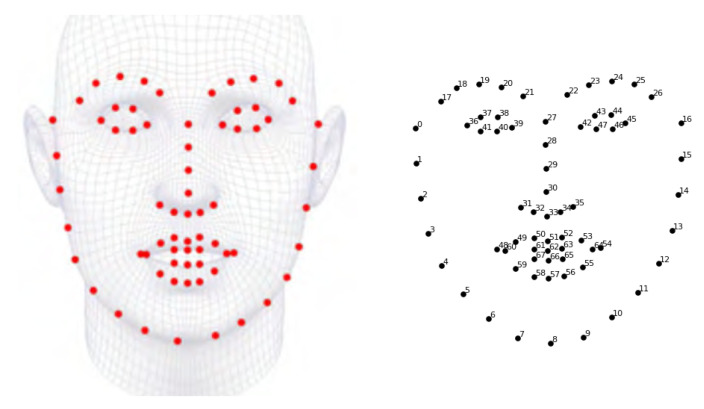
Illustration of the 68 face landmarks. Adapted from [27,28].68 Face Landmarks.

**No Recent Detection (Figure 4b):** The “No Recent Detection” mode serves to find new faces in the input images. It is called either when no face is actively being tracked or periodically to check if any new face appeared in the video stream. This part of the algorithm starts by applying a cascade classifier for frontal faces on the whole image. The frontal face detector is based on the general object detection framework described in [19] and uses a pre-trained model for frontal faces from [20] to extract the bounding box of faces in the input image. In order to prevent the algorithm from detecting already known faces, the input image is edited prior to exclude known faces. This is done by filling the bounding box of known faces with black pixels. After the application of the frontal face detector, a facial landmark detector based on an ensemble of regression trees, presented in [21], is applied to the image section of each detected face to estimate the position of facial landmarks. The output of the detector is an array with 68 elements which contain the *u* and *v* image coordinates of certain facial landmarks, shown in Appendix A, Figure A1. In the next step, the coordinates of the facial landmarks are used to calculate the yaw and roll angle of the face.

The head orientation is calculated on the basis of the *u* and *v* coordinates of certain facial landmarks, where *u* are horizontal image coordinates and *v* are vertical ones. The relative distance between facial landmarks allows for the estimation of the head orientation relative to the coordinate system of the camera. The roll angle is calculated from landmark number 42 and 39 which correspond to the tear ducts of each eye. The indices of the facial landmarks are illustrated in Appendix A, Figure A1. The roll angle is obtained with the equation:

(1) $$\mathrm{Roll}\;\mathrm{Angle}=\arctan\left(\frac{v_{42}-v_{39}}{u_{42}-u_{39}}\right).$$

Since the yaw angle cannot be obtained by this method, instead a substitute value is calculated which is proportional to the yaw angle. However, this value is suitable as a process variable for the robot controller and to determine if the user is oriented towards the robot arm. The substitute value is calculated using the landmarks number 27, 36, and 45 which correspond to the two outer corners of the eyes and the nose bridge. The substitute yaw angle is calculated with the equation:

(2) $$\begin{array}{c} \mathrm{Substitute}\;\mathrm{Yaw}\;\mathrm{Angle}=\frac{\sqrt{{\left(u_{45}-u_{27}\right)}^{2}+{\left(v_{45}-v_{27}\right)}^{2}}}{\sqrt{{\left(u_{27}-u_{36}\right)}^{2}+{\left(v_{27}-v_{36}\right)}^{2}}}-1. \end{array}$$

Finally, in the last step of the “No Recent Detection” mode a new entry is created in the detection data array for each detected face. The entries contain the bounding box of the face, the position of the facial landmarks, and the calculated head orientation.

**After recent detection (Figure 5):** The “After Recent Detection” mode serves to track faces. This mode is used when faces are “known” which corresponds to the detection data array having entries. This is the case either when a new face was found in the last iteration or when known faces are actively being tracked. The “After Recent Detection” mode performs several steps to track a known face which have to be repeated to track all known faces. In the first step, a subwindow area is extracted from the input image where the algorithm tries to redetect the face. The subwindow is derived from the last known bounding box of the face and is located at the same position but with a larger width and height. In this setup the subwindow is set to be 1.6 times larger in width and height than the old bounding box to account for movement. This approach achieves tracking of faces with the condition that faces stay in the subwindow area between frames. The size of the subwindow can be increased to improve tracking of faster moving objects which comes at the cost of an increased probability of false detections. The proposed methodology of utilizing subwindows of the input image allows for unambiguous tracking of multiple faces which are distinguished by their location in the camera image. Additionally, computational effort is reduced since smaller image areas are processed. After the extraction of the subwindow area, the frontal face detector is applied to redetect the known face. If the frontal face detector successfully redetects the face, the facial landmarks and head orientation are determined as described in “No Recent Detection” mode and the entry of the face in the detection data array is updated.

If no frontal face is detected, another detector for profile faces is used on the subwindow area to detect faces that are turned away from the robot arm. A profile face detector based on the general object detection framework from [19] with a pre-trained model for profile faces by [22] is used in this setup. The maximum turning angle for this detector is approximately 90°. If a profile face is found, the new bounding box is updated in the entry of the face. Facial landmarks are not calculated as the landmark detector is unreliable for faces that are not in a frontal view. The use of the profile face detector is documented and saved in the entry of the face as a boolean value. This value is used to tell the robot control that the position of the mouth cannot be tracked and to trigger the abort command for stopping the robot arm.

If both detectors fail to redetect the face in the subwindow, a counter is incremented that is saved in the detection data entry of the particular face. This counter is reset to zero when the face is redetected in a later iteration of the algorithm. However, if the counter reaches a certain threshold the particular face is considered lost and the entry of the face in the detection data is deleted. In this setup, the threshold is set to 10 which corresponds to 167 ms due to the 60 Hz rate at which the algorithm runs.

The detector-based tracking method proposed in this work offers robust tracking of faces and facial features for navigation and human robot interaction with a fast algorithm. In addition, it can distinguish different faces by their localization in the camera image which enables unambiguous tracking of faces. This feature prevents unpredictable situations where nearby standing people are mistaken for the user which can influence the robot’s trajectory and cause harm. A disadvantage of this algorithm is the limited capabilities in tracking fast moving objects, that leave the subwindow area between video frames. However, this scenario is very unlikely in assistive drinking as people of the target group are not capable of fast or wide ranging movement and the speed of the robot arm is very low.

#### 3.2.2. Distance Measurement and Projection

The proposed concept of the visual tracking system involves evaluation and correction of the distance sensor’s orientation for correct alignment and robust localization of the mouth. This method evaluates the orientation of the sensor using the location of facial features which are obtained from camera images, described in Section 3.2.1. The proposed method involves the projection of distance measurements to image coordinates for direct comparison of the location of distance measurements and facial features on a 2D-plane. The output value is the error of the alignment which is used to implement visual servoing for real-time sensor alignment correction by small movements of the robot arm. The projection of distance measurements involves transformation with the pinhole camera model and is presented below.

In the first step, a distance measurement is converted to the point *M* in 3D-space with regards to the coordinate system of the distance sensor. This coordinate system has its origin in the center of the receiver SPAD matrix and is oriented along the sides of the rectangle shaped sensor. The z-axis is oriented in the direction of measurement and the y-axis is oriented vertically. Due to the programmable receiver matrix, the FOV of the sensor is not always aligned with the main axis of the coordinate system. The deviation of the FOV in the vertical direction is called "Deviation" and must be taken into account. In this setup a Deviation of 0.5° was measured. The coordinates *X*, *Y*, and *Z* of the point *M* are obtained with the equation

(3) $$M=\left[\begin{array}{c} X\\ Y\\ Z \end{array}\right]=\left[\begin{array}{c} 0\\ \sin(Deviation)\cdot distance\\ \cos(Deviation)\cdot distance \end{array}\right].$$

In the next step, the point *M* is transformed into the coordinate system of the camera. The coordinate system of the camera has its origin in the camera sensor. The z-axis is oriented in the direction of image capture and the x-axis is oriented horizontally. The transformation Matrix [R|T] is derived from the spatial relationship of the distance sensor and the camera which is given by the assembly of the sensors. In this setup the spatial relationship is $\left[x_{axis},y_{axis},z_{axis}\right]=\left[-0.0175,-0.021,-0.022\right]$ in meters and [roll,pitch,yaw]=[−7.1,0,0] in degrees with regards to the camera coordinate system, as shown in Figure 2. The transformed point $M_{C}$ is calculated with the equation

(4) $$\left[\begin{array}{c} M_{C}\\ 1 \end{array}\right]=\left[\begin{array}{c} X_{C}\\ Y_{C}\\ Z_{C}\\ 1 \end{array}\right]=\left[R|T\right]\cdot \left[\begin{array}{c} M\\ 1 \end{array}\right],$$

where [R|T] describes the combined rotation and translation of the transformation matrix. Finally, $M_{C}$ is projected into the camera image to obtain the 2D-Point *m*. In this case, the pinhole camera model is used because the RealSense™camera of this setup performs internal processing to provide undistorted images. The projection is performed by multiplication of the point $M_{C}$ with the camera matrix *P*. The camera matrix *P* contains intrinsic properties of a camera which describe its unique FOV. In this case, it is provided by the factory calibrated RealSense™camera itself. The projection is performed with the equation

(5) $$m=\left[\begin{array}{c} u_{projected}\\ v_{projected}\\ 1 \end{array}\right]=\frac{1}{Z_{C}}\cdot P\cdot M_{C}=\frac{1}{Z_{C}}\left[\begin{array}{ccc} f_{x} & 0 & c_{x}\\ 0 & f_{y} & c_{y}\\ 0 & 0 & 1 \end{array}\right]\cdot \left[\begin{array}{c} X_{C}\\ Y_{C}\\ Z_{C} \end{array}\right],$$

where $f_{x}$, $f_{y}$, $c_{x}$, and $c_{y}$ are the cameras intrinsic calibration parameters. The output of this transformation is the point *m* which corresponds to the location of the object that the distance sensor is pointed at in the camera image. This point enables direct comparison of the location where the distance sensor is pointed at and the location of the mouth on the 2D plane of the camera images. Therefore, enabling the sensor fusion algorithm to evaluate the alignment of the distance sensor towards the mouth.

#### 3.2.3. Sensor Alignment Estimation

**Figure 6:**
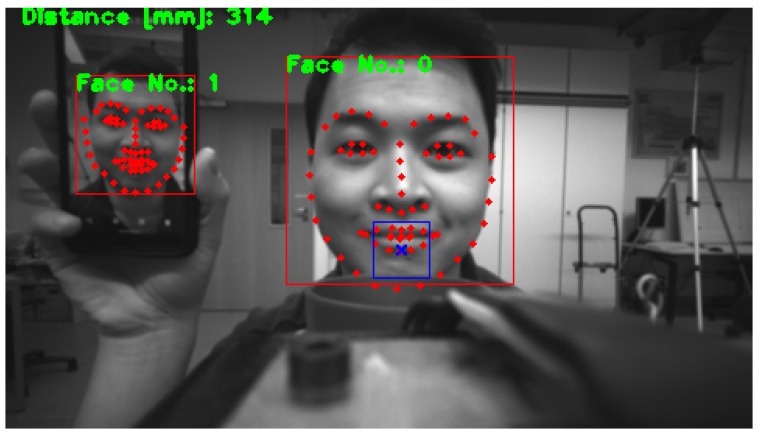
Visual representation of the input data for the sensor alignment estimation. The picture shows a camera image with an overlay of the input data. Two faces are being tracked unambiguously which are labeled as Face No.: 0 and 1. The red dots illustrate the position of the facial landmarks. The projection of the distance measurement is illustrated by the blue cross which is framed by a blue rectangle. The rectangle symbolizes the field of view of the distance sensor. In this picture the distance sensor is not properly aimed at the upper lip.

The sensor alignment estimation is a crucial step of the proposed concept to evaluate and ensure that the distance sensor is correctly oriented for robust localization of the mouth. It compares the location of a distance measurement with the location of the upper lip in the image plane to determine the error of the distance sensor alignment. Afterwards the error value is send to the robot controller to automatically correct the orientation using a visual servoing controller. The inputs of this algorithm are the location of the facial features, ${\vec{u}}_{landmarks}$ and ${\vec{v}}_{landmarks}$, and the projection of distance measurements, $u_{projected}$ and $v_{projected}$. Figure 6 illustrates the input data of the sensor alignment estimation.

The location of the upper lip in the camera image is given by the image coordinates of the facial landmark number 51. The comparison is performed by subtraction of the image coordinates of landmark number 51 and the projected distance measurement. The output of the calculation is the horizontal and vertical error Δu and Δv of the alignment and is calculated with the equation

(6) $$\begin{array}{c} \Delta u=u_{landmark51}-u_{projected}\\ \Delta v=v_{landmark51}-v_{projected}. \end{array}$$

The output values Δu and Δv are sent to the robot controller which moves the robot arm to correct the aim of the distance sensor. This part of the robot control algorithm is described in Section 3.3 and called sensor alignment correction.

### 3.3. Robot Control Algorithm

The proposed method for robust localization of the mouth in assistive drinking involves a two step process consisting of estimating the misalignment of the distance sensor and correcting the sensor alignment. While the first step is handled by the algorithm described in Section 3.2.3, the correction of the sensor alignment is handled by a controller which adjusts the robot arm. In this work a robot control algorithm is proposed which simultaneously corrects the alignment of the distance sensor and autonomously navigates the robot-handled cup to deliver the drinking cup. The delivery of the cup involves the navigation of the robot-handled drinking cup from an arbitrary starting position to the mouth of the user where physical contact is made between the lower lip and the cup rim. The two tasks of the robot control algorithm are described in the following.

**Sensor Alignment Correction:** The input of the sensor alignment correction are the horizontal and vertical error Δu and Δv as image coordinates. Our approach uses visual servoing with a closed-loop controller to minimize Δu and Δv by moving the end-effector of the robot arm where the tracking system is assembled. The controller is implemented with two PID controllers which control one DOF each. The control variables are mapped to the vertical movement and rotation around the yaw axis of the robot’s end-effector. These DOFs were chosen because rotation around the pitch and roll axis would result in spillage of the cup’s content. The control variables are updated with a 60 Hz rate in correspondence to the frequency of the Sensor Alignment Estimation. This visual servoing based approach enables precise alignment of the distance sensor for robust localization of the mouth with a simple control structure. As the navigation algorithm is specifically designed for this system, the sensor alignment correction is performed simultaneous to the delivery of the cup.

**Delivery of the Drinking Cup:** A navigation algorithm for the delivery of the cup is proposed which uses closed-loop controllers based on visual servoing principles. The inputs of the navigation algorithm are the coordinates of facial landmarks, the orientation of the head and the distance measurement of the TOF sensor. The starting point of the navigation is an arbitrary pose where the cup is positioned in front of the user and the visual tracking system is oriented towards the user. It is mandatory for the user’s face to be in the FOV of the camera. However, the distance sensor can be misaligned at the start and will be aligned once the navigation is started. The target, shown in Figure 2 by the transparent face and black dot, is to position the cup rim on the mouth’s lower lip. Since the lower lip is occluded by the cup rim, a capacitive sensor is incorporated from our previous work [15] which is assembled on the cup rim to detect physical contact. Compared to a force sensitive resistor that requires an activation force which can exceed a few newtons, the measuring principle of the capacitive sensor allows the detection of physical contact nearly without contact force.

**Figure 7:**
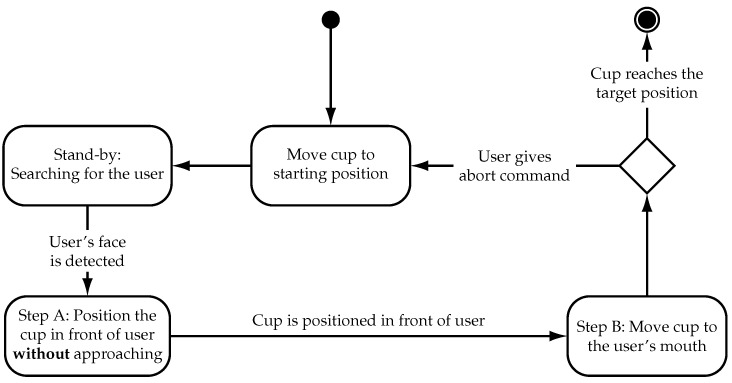
State machine of the navigation algorithm to perform the delivery of the cup.

The proposed navigation algorithm is implemented as a state machine which performs the cup delivery in two steps. In the first step the cup is aligned directly in front of the user’s face in order to move the cup in a straight line towards the user’s mouth in the second step. This approach serves to reduce the complexity of the robot’s movement when the cup is moved towards the user to make the process more comprehensible and predictable for the user. In addition, an exit condition is implemented that is triggered by the abort command, described in Section 3.2.1. The state machine shown in Figure 7 is described below. The initial state is “Move cup to starting position”.

**Figure 8:**
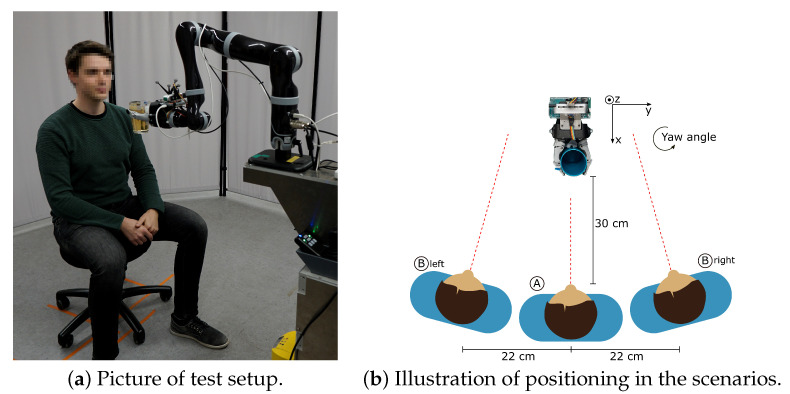
Illustration of the test setup and the position of the robot and subject for the different scenarios. Position A is used in scenario one and two. Position B is used in scenario three. The dashed red lines represent the heading direction.

The cup delivery process begins after filling and grasping of the cup. In the first step, the cup is moved to a predefined starting pose which is approximately 30 cm in front of the user. This position allows the visual tracking system to detect the user which is necessary to perform the rest of the cup delivery. The setup is illustrated in Figure 8.

Stand-by, searching for the user: After reaching the starting position, the robot goes into a stand-by mode and uses the visual tracking system to search for faces. Detected faces are evaluated by the size of the bounding box to roughly estimate the distance. Small faces are rejected as they are too far away to interact with.

Step A, positioning of the cup: If a suitable face is detected, step “A” of the cup delivery is started. At this point, the sensor alignment correction is engaged for the rest of the interaction which ends when the cup is delivered or when the process is aborted. Step “A” serves to position the cup in front of the user’s face and align the gripper with the heading (yaw axis) direction of the user. This is accomplished using only one PID controller in conjunction with the sensor alignment correction which is running simultaneously. This PID controller takes the substitute yaw-angle, described in Section 3.2.1, as an input and moves the robot’s gripper sideways to reduce the relative yaw-angle between the tracking system and face of the user to zero. Therefore, the gripper is aligned with the heading direction of the user. The resulting motion is a combination of sideways movement to align the gripper, rotation around the yaw-axis to turn the tracking system towards the user and vertical movement to position the cup at the correct height.

Step B, move to user: When the cup is aligned and positioned in front of the user, step “B” of the cup delivery is started where the cup is moved towards the mouth. In this step, the orientation of the cup is locked as the cup is already oriented correctly to establish physical contact. The speed at which the cup is moved towards the user is regulated by the distance between the cup and mouth. This approach allows for greater speed at larger distances and slower speeds in the proximity of the user to prevent harmful contact force in the event of an accident. At a distance of 30 cm the movement speed is approximately 3 cm/s which is reduced to 1 cm/s in the final centimeters of the delivery. Additional controllers are not required, as positioning errors are automatically corrected by the sensor alignment correction. The target position is reached and the cup is stopped when physical contact is detected by the capacitive sensor on the cup rim. Physical contact is detected when the capacitance of the sensor suddenly changes.

Abort Command: During step “B” of the cup delivery as the cup moves towards the mouth, the user can abort the process at any time. In this case, the cup is returned to the starting position immediately and the robot goes into the stand-by mode. The abort command is implemented with two conditions. The first is linked to the boolean value from the profile face detector described Section 3.2.1 and is triggered by a head turn motion of the user. The second condition is triggered when the face of the user is deleted from the detection data array, described in Section 3.2.1, which corresponds to either an error in the face tracking algorithm or occlusion of the user’s face.

The proposed navigation algorithm based on visual servoing methods enables the delivery of the cup. The algorithm was successfully implemented on the robotic system and integrated with the visual tracking system. Initial tests in the development cycle showed that the system is capable of delivering a drinking cup and establishing physical contact with the lower lip. Evaluation of the proposed system focuses on the ability of the system to position the cup at the mouth reliably as well as the usability of the safety features. These evaluations are subject of the next chapter.

## 4. Experimental Setup

After successful implementation of all components, initial tests in the laboratory were conducted with success. However, important specifications regarding reliability and safety need to be determined for a proper evaluation of the proposed system. The evaluation focuses on two properties in particular which are determined in two experimental studies. These are the reliability of the system to deliver the drinking cup, as well as the reliability and usability of the abort command. These properties are chosen specifically as they are essential to the overall system. This is due to the fact that correct positioning of the cup is mandatory for successfully performing assistive drinking. Furthermore, unreliable delivery of the cup quickly leads to user frustration and facilitates accidents. In addition, reliable detection of the abort command is essential for safe human robot interaction as it enables the user to control the robot, which is especially important in unpredictable situations.

Two experimental studies were conducted which featured able-bodied test subjects. In the first study the success rate of correctly positioning the cup in the cup delivery is measured by repeatedly performing the cup delivery with several test subjects. Additionally, three scenarios were defined which challenge different aspects of the system. In the second study, the detection rate of the abort command is measured with several test subjects. This is done by repeated execution of the head gesture for triggering the abort command. At the same time, the required turning angle for triggering the abort command is measured with an external reference systems to evaluate the required range of motion. The experimental setup of the studies is described below.

### 4.1. Subjects

Nine able-bodied test subjects participated in the two studies described below. The able-bodied subjects had no known limitations in terms of range of motion. Six of them were male and three female. It was hypothesized that certain facial features which obstruct the view of the face influence the performance of the face tracking algorithm. For this reason, the subjects were chosen based on facial appearance and assigned to one of three equally sized groups. These groups are: (1) people without obstructions of the face (the control group) (2) people with glasses (3) people with beards. Their age ranged from 22 to 58 years (mean ± standard deviation: 35.22 ± 13.00 years). All subjects gave their written consent to the experiment and were instructed in detail about the procedure. This study was approved by the ethics committee of the German Association of Social Work (DGSA).

### 4.2. Success Rate of the Cup Delivery Process

Correct positioning of the cup and high reliability of the proposed robotic system are important for intuitive and safe execution of assistive drinking. These properties are a result of accurate localization of the mouth and navigation which are necessary for correct positioning of the cup at the lips of the user. Furthermore, high reliability in performing the autonomous task is desired for an easy and intuitive experience.

In this study, the success rate of performing the cup delivery is measured as an indicator to evaluate the aforementioned properties. The test involves multiple executions of the cup delivery with each of the nine test subjects. Since different user behavior can influence the performance of the cup delivery, three test scenarios are proposed to evaluate the success rate with different actions of the user. These scenarios are: ideal conditions where the user stays still, movement of the user while the cup is delivered to the mouth, and misalignment of the user at the start of the cup delivery. Each test run, which consists of one execution of the cup delivery, is evaluated by four conditions to determine if the test run was successful or not. The result (successful or not) and the cause of failure were documented. The four conditions are:

- The capacitive sensor on the cup rim touches the lips of the subject and the cup was positioned at the target position described in Section 3.2 and shown in Figure 2
- Physical contact was established gently with minimal contact force which did not push the subject backwards
- The delivery was not aborted prematurely by loss of tracking
- The delivery was not aborted by a false-positive abort command

The cup delivery is executed 16 times per test subject and scenario for a total of 48 test runs per subject. The test setup is illustrated in Figure 8.

The test runs were conducted as follows: The subject sits on a stool in front of the robot arm. The robot-handled cup rests in the starting position and the robot is in the stand-by mode. For this study, a start signal has to be given by the test instructor for the robot to start the cup delivery. The subjects were instructed to hold their head straight, keep a neutral facial expression, and not talk during the cup delivery to ensure consistent conditions in all test runs. In addition, movement should be avoided except for scenario 2. The tests were performed in a laboratory with regular room lighting conditions. Strong directional lights such as sun rays and spotlights were avoided. The test runs were performed according to the scenarios described below:

(1) Ideal Condition: The first scenario aims to test the system in ideal conditions. The subject is positioned directly in front of the robot (position A) and is instructed to move as little as possible.
(2) Arbitrary Movement: The second scenario aims to simulate arbitrary movement of the user during the cup delivery. This scenario is particularly challenging for the tracking system because a moving target has to be tracked which requires precise real-time localization. In this scenario, the subject sits in position A. When the cup delivery is started by the test instructor, the robot performs the positioning and alignment step of the navigation algorithm. Afterwards when the cup is moving towards the mouth, the subject moves their head towards or away from the cup while keeping their shoulders stationary. The constrained head motion serves to simulate the range of motion of people with tetraplegia. This test is performed eight times with the subject moving towards and eight times with the subject moving away from the cup.
(3) Misalignment at the Start: In the third scenario misalignment at the start of the cup delivery and by this extend a suboptimal start position is simulated to test the performance of the sensor alignment correction and head orientation estimation. In this scenario, the subject is situated as far right (position B right) or left (position B left) of the cup as possible while the face is still visible in the camera image. Additionally, the gaze direction of the subject is past the tracking system. After the start signal is given the test is conducted as in scenario 1. This test is conducted eight times for the right side and eight time for the left side.

### 4.3. Detection of the Abort Command

The ability to reliably stop an autonomous robotic system at will is an important component to achieve safe human robot interaction in assistive drinking. Therefore, the reliable detection of the proposed abort command is mandatory for safe operation of the robotic system. This study serves to determine the reliability of the proposed abort command by measuring the detection rate for nine test subjects. In addition, the required range of motion to activate the abort command is evaluated by measuring the turning angle at which the abort command is activated by the test subjects. The required range of motion is an important indicator for the accessibility of the abort command because most people with tetraplegia also experience restricted range of motion.

**Figure 9:**
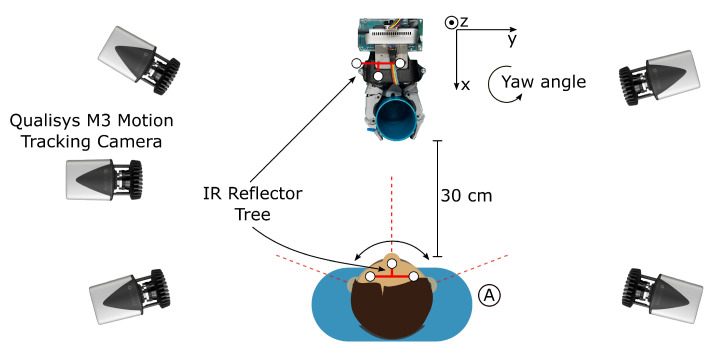
Test setup to measure the required angle to trigger the abort command. The red dashed lines represent the heading direction.

The reliability and required range of motion of the abort command are experimentally evaluated with nine test subjects by repeated activation of the abort command. In this test setup, the robot arm is parked in the starting position and the visual tracking system is turned on. The test subject sits in front of the robot arm. An external camera-based motion tracking system from Qualisys [23] is used to determine the rotation angle of the head in regards to the visual tracking system by measuring the pose of both. The external reference system uses IR reflector trees which are worn as a headband by the subject and mounted on the end-effector of the robot. The external reference systems consists of five Qualisys M3 cameras that are placed around the robot. The setup is shown in Figure 9.

The test was conducted as follows: the subject sits in front of the robot (position A). In the first step the subject is instructed to look straight at the camera. This orientation is recorded as the starting pose. The subject is then instructed to turn their head to the left and then to the right as far as possible without moving their shoulders. This restriction serves to simulate the range of motion of people with tetraplegia who, in most cases, can only move their head with the neck. The head orientation is recorded when the abort command is triggered at some point during the head turn. The subjects are instructed to turn their head slowly with a speed of approximately 10°/s to measure the angle at which the abort command is activated accurately. This procedure is repeated 20 times for right turns and 20 times for left turns. In total 40 head turns are performed per subject.

## 5. Results

The two experiments described above were performed to determine the reliability of the system for delivering the drinking cup as well as the reliability and usability of the abort command. The results of the tests are the success rate for the cup delivery, the detection rate of the abort command, and the required range of motion to activate the abort command which are described in the following chapters.

### 5.1. Success Rate of the Cup Delivery

The cup delivery was performed 16 times per test subject for a total of 432 test runs. 430 of the test runs were successful in delivering the drinking cup to the mouth of the user. The complete result is shown in Table 1.

In total, the results show an overall success rate of 99.54%. The worst overall success rate of any scenario is 99.31% and that of any subject is 97.83%. In this study, only two unsuccessful test runs occurred which happened in different scenarios and with different subjects. The unsuccessful tests are described in the following:

**Error Case** **1:**  Subject 5 Scenario 3: The visual tracking system lost track of the user. The cup delivery was aborted and the robot arm returned to its starting position.
**Error Case** **2:**  Subject 7 Scenario 2: The user tilted the head backwards slightly. The robot-handled cup made first physical contact with the chin of the subject and did not stop after contact was made. Consequently the subject was pushed backwards. The subject did not feel any pain and was not injured, due to the low speed setting of the robot and the safety precautions of the test.

### 5.2. Reliability of the Abort Command

The evaluation of the abort command provides the detection rate and the required turning angle to trigger the command. All head turns performed by the subjects were detected correctly as an abort command by the tracking system. The abort command was always triggered by detection with the profile face detector. The result is a detection rate of 100%.

**Figure 10:**
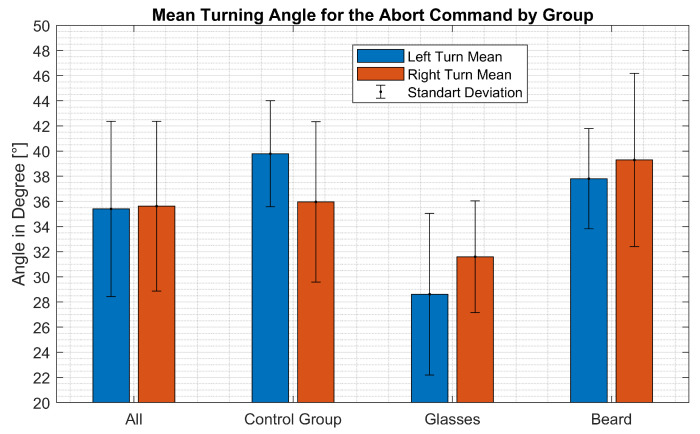
Bar graph of the average turning angles required to trigger the abort condition per group. The blue bars represent the mean value for left turns and the red bars for right turns.

The Figure 10 illustrates the average turning angle at which the abort command was triggered per group. The average angle and standard deviation for each group is calculated for both directions. On average the test subjects had to turn their head by approximately 35.5° in either direction to trigger the abort command with a standard deviation of approximately 6.8° degrees. The measured turning angles show no significant difference between left and right turns with an average difference of 0.21° (*p* = 0.769). However, significant differences could be determined between the groups. On average, subjects with glasses triggered the abort command at a smaller angle than the control group (absolute difference: L: 11.17° R: 4.35°, *p* < 0.001). The subjects with beards had to turn their head further than the control group (absolute difference: L: 1.98° R: 3.33°, *p* < 0.01). The result of the individual subjects are listed in Table A1.

## 6. Discussion

In this work two studies were conducted to evaluate the visual sensor fusion based autonomous robotic system for assistive drinking. The result of the studies provide insight into the performance, reliability and usability of the proposed system, which is discussed in the following section.

### 6.1. Performance of the Cup Delivery

In total, the results of the first study indicate a high success rate for the delivery of the cup. It can be concluded that the proposed system has a high reliability in tracking the user and navigating the cup to the desired position at the user’s mouth. Furthermore, the results show no significant performance difference for any scenario or subject group that was tested. This indicates that the proposed system achieves robust localization and tracking of the mouth even with arbitrary movement of the user which is beneficial for robust daily use. Furthermore, the system is capable of navigating the cup despite suboptimal positioning in regards to the starting position of the cup. In addition, wearing glasses or having a beard did not have a measurable impact on the performance of the system in the tested scenarios.

In conclusion, the proposed system is fully capable of delivering a cup to a user who is moving the head or who is misaligned to the robot arm at the start of the procedure. In addition, the proposed system is also capable of interacting with people who have a beard or who wear glasses. However, the facial appearance can vary in many more parameters like age, skin color, and gender as well as facial features like tattoos, piercings, scars, and facial paralysis which can have an effect on the robustness of the face detector and ultimately the entire tracking system. The influence of these parameters is highly dependent on the dataset the face detector is trained on and cannot be evaluated from the given data due to the small number of test subjects.

#### 6.1.1. Implication of the Error Cases

An evaluation of the error cases that occurred during the study to evaluate the success rate of the cup delivery allow for a more detailed assessment of the proposed system. The two error cases are described and analyzed below.

In the first error case, the tracking system lost track of the user and stopped the procedure as intended. However, the subject’s face was visible in the video stream of the tracking system. An explanation for the error could be a reflection in the glasses of the test subject which obscured the eyes. During development of the face tracking algorithm the frontal face detector was shown to be sensitive to occlusion of the eyes. It was observed that the frontal face detector was unreliable in detecting faces with covered eyes while this issue does not appear on faces with covered mouth or nose.

**Figure 11:**
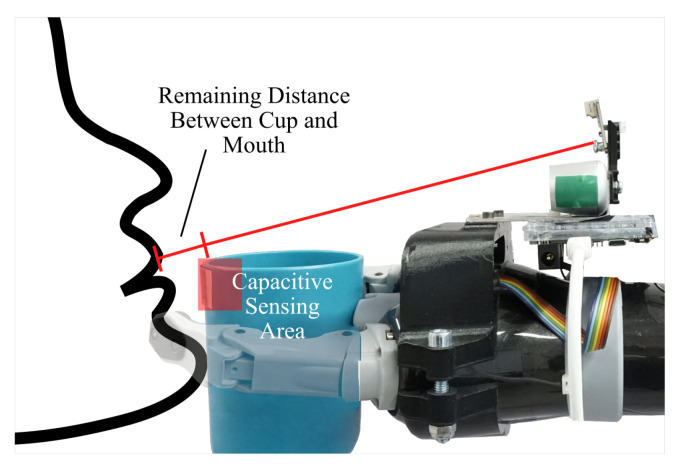
Schematic illustration of the second error case.

The analysis of the second error case suggests a blind spot of the sensor system that leads to an error where the robot moves unintentionally. In this incident the subject tilted the head backwards which moves the chin forward and causes it to be the first point of contact with the robot-handled cup. The area where the chin touches the cup is lower than the target contact point where the lips are suppose to touch the cup rim. Therefore, physical contact was not detected since the capacitive sensor does not extend that far down. Therefore the robot control continued to move the robot forward pushing the user’s head backwards. Furthermore, also the tracking system was not able to recognize that the cup was in contact with the user. This is because the tracking system, by design, is aimed at the upper lip which was still a few millimeters away from the cup due to the head pose. This circumstance is illustrated in Figure 11. In summary, the current implementation of the robotic system is not able to detect this error because it occurs in a blind spot. Furthermore, the robot-handled cup is physically not able to establish physical contact with the lips due to collision with the chin when the the user’s head is tilted backwards. Also, tilting the cup to clear the chin is not feasible as the cup’s content could spill. Therefore, we propose two solutions to detect backwards tilting of the head and subsequently abort the cup delivery process. The first solution is to increase the sensing area of the capacitive sensor to the whole front side of the cup. This solution enables the detection of contact in a larger area which would close the blind spot. A second solution is the integration of a head pose estimation algorithm, as described in the work of [24], in the face tracking algorithm. The estimated head pose is then used to stop the cup delivery in case the user tilts their head backwards.

### 6.2. Reliability of the Abort Command

The second test to evaluate the abort command showed a detection rate of 100% regardless of facial appearance. Therefore, it can be concluded that this implementation of the abort command can be activated very reliably by turning the head. However, the measurements suggest that a certain range of motion is required for the activation of the abort command. The required range of motion is described below.

### 6.3. Required Movement Range

The study for evaluating the abort command shows that the proposed approach achieves reliable detection of the head turning gesture. However, the measurements show that the angle at which the abort command is triggered can vary between multiple executions. Therefore, a certain minimum turning angle of the head is required for reliable activation of the abort command as small head turns may not be detected. As a result, the user needs to have a certain range of motion to use it reliably. Significant differences in the turning angle at which the abort command was triggered were observed between the groups of subjects. It is concluded from these results, that wearing glasses reduces the required turning angle of the abort command compared to the control group without facial obstructions. Furthermore, having a beard increases the required turning angle slightly compared to the control group. One possible explanation is the occlusion of the eyes by reflections on the glasses, as described in Section 6.1.1.

Based on this data, the required range of motion is dependent on facial appearance. However, the largest values were observed in the group with beards. These values suggest a maximum turning angle of 53.07° (right turn mean of beard group plus 2× standard deviation) to trigger the abort command with 97.5% security. These figures suggest that a maximum range of motion of 106.14° is required for reliable activation of the abort command. Able-bodied people have a range of motion of 160°, according to standard values [25]. Thus, the able-bodied participants of our study are able to use the abort command without restrictions.

However, the range of motion of people with tetraplegia or spinal cord injuries can vary drastically. A study by LoPresti et al. [26] measured the range of motion of the head. Multiple subjects participated which suffered from different physical disabilities related to paralysis and spinal cord injury. The study showed a total range of motion for the axial rotation of the head of 85,6° with a standard deviation of 39.7°. These values indicate that 49% of participants from [26] have the required range of motion of 106.14° to use the abort command with 97.5% security and operate the robot safely in every scenario.

## 7. Conclusions

This work presents a visual sensor fusion based autonomous robotic system for navigation tasks in assistive drinking. The proposed visual tracking system is able to localize the mouth of a person reliably by fusion of data from a regular camera and a TOF single-point distance sensor. In contrast to systems that use distance sensors exclusively, this approach is able to determine where the sensor is pointed at and correct the alignment to aim the sensor at the intended object. This prevents a common measurement error caused by misalignment. Additionally, the tracking system enables unambiguous tracking of multiple faces and detection of the head orientation for safe human robot interaction. Finally, a navigation algorithm is proposed that utilizes the sensor fusion data to autonomously navigate a robot arm in a cup delivery process. This process involves the navigation of a robot-handled cup from a starting position to the mouth of a human user, while establishing physical contact.

A first prototype with the Kinova^®^Jaco^®^robot arm was integrated successfully. An experimental evaluation with nine able-bodied test subjects showed that this system can reliably localize and track the user’s mouth to navigate the robot arm. Furthermore, this system was able to establish physical contact between the robot-handled cup and the user’s lips with minimal contact force. The study also suggests that the proposed system is able to adapt to difficult conditions such as movement of the user and misalignment at the start of the cup delivery. The detection of an abort command for stopping the robot was evaluated and showed to be very reliable, provided the user has a range of motion of at least 106°.

Finally, a sensor fusion algorithm was demonstrated that enables a robot to localize the mouth by combining camera images and distance measurements. In combination with a navigation algorithm, it is able to establish physical contact between a robot-handled cup and the user’s lips. These skills are fundamental for assistive robotics and human robot interaction with physical contact. Therefore, it can serve as orientation for other assistive robotics applications with physical contact or could even be adopted in these applications.

## 8. Future Work

This work demonstrates a sensor fusion algorithm to evaluate the alignment of a single-point distance sensor with a camera which allows for precise alignment of the distance sensor and reliable localization. This concept enables a variety of other use cases that are based on measurements with distance sensors, e.g pick-and-place tasks and other assistive robotics applications like eating.

The studies in this work were conducted with able-bodied test subjects due to the pandemic situation. However, the results suggest that the range of motion influences the reliability of the proposed abort command. Therefore, future research will focus on further evaluation of the proposed robotic system in a study with people affected by tetraplegia. Furthermore, additional computer vision-based features will be investigated to improve the safety of the system. For example, a head orientation estimation algorithm which enables the tracking system to estimate the heading direction of the face for all three axis. This additional data is useful in two ways: prevention of a potentially harmful situation where the user’s tilts their head backwards and enabling the activation of the abort command at a specific turning angle for user’s with restricted range of motion. Additionally, different face detectors will be assessed to evaluate and improve the robustness of the face detector system to different facial appearances.

Future work will also conduct further studies including people with different physical disabilities to further evaluate the usability, target group, accessibility requirements, and performance in different real world scenarios.

## Figures and Tables

**Table 1:** The number of successful cup deliveries and the success rate for each subject and scenario are listed. In addition, the total success rate for each subject and each scenario as well as the overall success rate is given.

Subject Number		Scenario			Success Rate of Each Subject
		1	2	3	
1	Control Group	16 100%	16 100%	16 100%	48 100%
2		16 100%	16 100%	16 100%	48 100%
3		16 100%	16 100%	16 100%	48 100%
4	with Glasses	16 100%	16 100%	16 100%	48 100%
5		16 100%	16 100%	15 93.75%	47 97.83%
6		16 100%	16 100%	16 100%	48 100%
7	with Beard	16 100%	15 93.75%	16 100%	47 97.83%
8		16 100%	16 100%	16 100%	48 100%
9		16 100%	16 100%	16 100%	48 100%
**Success Rate of** **each Scenario**		144 100%	143 99.31%	143 99.31%	430 99.54%
			

## Data Availability

Publicly available datasets were analyzed in this study. This data can be found here: https://github.com/pietertry/Visual-Sensor-Fusion-based-Autonomous-Robotic-System (accessed on 3 August 2021).

## Abbreviations

The following abbreviations are used in this manuscript:

MDPI	Multidisciplinary Digital Publishing Institute
ADL	Activities of Daily Living
TOF	Time of Flight
DOF	Degrees of Freedom
WMRA	Wheelchair Mounted Robot arm
HMI	Human Machine Interface
GUI	Graphical User Interface
BMI	Brain Machine Interface
ROS	Robot Operating System
FOV	Field of View
SPAD	Single Photon Avalanche Diode
DGSA	German Association of Social Work

## Appendix A

**Table A1:** Average turning angle for triggering the abort condition per subject.

		Mean Angle [°]		Standard Deviation [°]	
Average Values		Right Turn	Left Turn	Right Turn	Left Turn
Control Group	1	−38.67	42.58	4.96	2.88
	2	−38.66	38.20	3.58	2.83
	3	−30.77	38.26	6.61	5.20
with Glasses	4	−28.17	24.05	3.43	7.68
	5	−35.50	33.92	3.46	3.54
	6	−31.13	27.88	2.96	2.19
with Beard	7	−30.92	32.94	3.20	2.39
	8	−42.35	40.00	3.93	1.53
	9	−44.78	40.47	1.86	1.94
**Average Values**					
**Overall**		−35.61	35.40	6.75	6.96
**Control Group**		−35.95	39.78	6.38	4.21
**with Glasses**		−31.6	28.62	4.44	6.43
**with Beard**		−39.29	37.80	6.89	3.99
